# Discovery of a Novel *Bacillus* sp. JO01 for the Degradation of Poly(butylene adipate-*co*-terephthalate)(PBAT) and Its Inhibition by PBAT Monomers

**DOI:** 10.4014/jmb.2408.08051

**Published:** 2024-11-25

**Authors:** Jinok Oh, Nara Shin, Suwon Kim, Yeda Lee, Yuni Shin, Suhye Choi, Jeong Chan Joo, Jong-Min Jeon, Jeong-Jun Yoon, Shashi Kant Bhatia, Yung-Hun Yang

**Affiliations:** 1 Department of Biological Engineering, College of Engineering, Konkuk University, Seoul 05029, Republic of Korea; 2 Department of Chemical Engineering, Kyung Hee University, Yongin-si 17104, Republic of Korea; 3 Green & Sustainable Materials R&D Department, Korea Institute of Industrial Technology (KITECH), Cheonan-si 31056, Republic of Korea; 4 Institute for Ubiquitous Information Technology and Application, Konkuk University, Seoul 05029, Republic of Korea

**Keywords:** Degradation, degradable bacteria, bioplastic, Poly(butylene adipate-*co*-terephthalate), environmental sustainability

## Abstract

Poly(butylene adipate-*co*-terephthalate) (PBAT) is a type of biodegradable plastic composed of both aliphatic and aromatic hydrocarbon polymers, which grants it the advantages of processability and flexibility along with increased interest. Studies have suggested that PBAT biodegradation mechanisms involve enzymatic breakdown by lipases. Our initial efforts in this study were therefore focused on identifying a novel PBAT-degrading bacterial strain with high degradation activity. Nine bacterial strains from various sources were screened and assessed for their ability to degrade PBAT. *Bacillus* sp. JO01 strain, exhibiting high similarity (99%) with *Bacillus toyonensis* BCT-7112, demonstrated superior PBAT degradation activity under various temperature conditions from 25 to 42°C. Time-dependent PBAT degradation by *Bacillus* sp. JO01 indicated a maximum yield at 30°C, reaching 66% of film degradation measured. Besides PBAT, the strain showed degradability on PCL, PHB, and PBS. Physical characterization of the degraded PBAT films via scanning electron microscopy revealed that surface alterations such as cracks were reduced, as was the molecular weight. *Bacillus* sp. JO01 did not consume PBAT monomers, such as adipic acid (AA), 1,4-butanediol, and terephthalic acid (TPA). However, AA and TPA showed inhibitory effects on the degradation of PBAT films by *Bacillus* sp. JO01, resulting in 30% inhibition of degradation at 16 mM of AA and at 32 mM of TPA. This study highlights *Bacillus* sp. JO01 as a superior strain for PBAT degradation and suggests that PBAT monomers have an inhibitory effect on the degrading strains, which is an important consideration for the bulk degradation of bioplastics.

## Introduction

Polymers, particularly plastics, are the most extensively used materials in daily life. Global plastic production has surged from 2 million tons in 1950 to 359 million tons in 2019, with projections indicating that the cumulative global plastic production volume will reach 26 billion tons by 2050 [1].

The increased production of plastics from petroleum underscores the need for sustainable alternatives that are sourced from renewable resources to minimize unfavorable environmental effects. Effective management of used plastic materials involves recycling as a solution. However, the process of selecting and cleaning recyclable plastics is expensive, and foreign substances may be mixed in the processing stage, resulting in inferior properties, such as strength and elasticity, which limit the application of recycled plastics. Another approach to reducing plastic residue is the utilization of biodegradable plastics or the biodegradation of used plastics [2]. Although bioplastics are an attractive alternative, timely degradation of bioplastics is also required for their utility; therefore, this study focused on identifying superior players in bioplastic degradation.

Biodegradation of a material refers to any physical or chemical change induced by the activity of micro-organisms [3]. Both natural and synthetic plastics undergo degradation under the influence of microorganisms, such as bacteria, and fungi [4]. These microorganisms are widely distributed in soil and compost [5]. More than 90 types of microorganisms, including aerobes, anaerobes, photosynthetic bacteria, archaebacteria, and lower eukaryotes, play crucial roles in bioplastic biodegradation and catabolism. The biodegradation of polymers involves three sequential steps: (a) attachment of microorganisms to the polymer surface, (b) utilization of the polymer as a carbon source, and (c) polymer degradation. Microorganisms adhere to polymer surfaces and initiate degradation by secreting enzymes that extract energy for growth [6]. This breakdown leads to the conversion of large polymers into monomers and oligomers, which are characterized by their low molecular weight. Certain oligomers may permeate the internal environment of the microorganism and be assimilated after diffusion.

This study focuses on poly(butylene adipate-*co*-terephthalate) (PBAT), a petroleum-based but biodegradable plastic. The market for PBAT is expected to grow to 112 million tons by 2025. The most important features of PBAT are its flexibility and processability. Owing to its excellent flexibility, it is used in disposable, shopping, and standard garbage bags. To manage and meet this increasing global demand for biodegradable plastics, it is important to find a way to process these materials quickly [7]. PBAT was prepared by blending 1,4-butanediol, AA, and TPA. Among its monomers, 1,4-butanediol and TPA are responsible for the rigid regions, while 1,4-butanediol and AA regulate the flexibility of the polymer [8].

Several microorganisms have been reported to degrade PBAT (Table 1). *Stenotrophomonas* sp. YCJ1 isolated from farmland soil showed 10.14 wt% of PBAT degradation at 37°C for 5 days [9]. *Peribacillus frigoritolerans* S2313, isolated from the soil of an agricultural cotton field in Shihezi, China, can degrade 3.98% of PBAT at 28°C for 7 days [10]. *Bacillus pumilus* was isolated from soil, and this strain reached 6 wt% of PBAT when cultivated at 30°C for 10 days [11].

In this research, we identified a strain with good PBAT degradability and studied its properties. The activity of the strain we used was determined for comparison with various other strains that degrade PBAT. High-pressure liquid chromatography (HPLC) and a gas chromatography flame ionization detector (GC-FID) were used to monitor the effect on degradation of PBAT monomers, which could be the main end products of degradation. We investigated the consumption of monomers by *Bacillus* sp. JO01, and based on these results, confirmed that the monomers acted as inhibitors of PBAT degradation.

## Materials and Methods

### Chemicals

All chemicals used in this study were of analytical grade. Chloroform and dichloromethane (DCM) were procured from Junsei Chemical Co. (Japan). Polylactic acid (PLA) and polycaprolactone (PCL) were purchased from Sigma-Aldrich (USA). Polyhydroxybutyrate (PHB) pellets were obtained from Goodfellow Cambridge Ltd.(UK). Polybutylene succinate (PBS) pellets were sourced from ANKOR Bioplastics Co., Ltd. (Republic of Korea). PBAT was obtained from BASF SE (Germany). Poly-3-hydroxybutyrate-*co*-4-hydroxybutyrate (P(3HB-*co*-4HB)) pellets were procured from CJ (Republic of Korea).

### Preparation of Bioplastic Films

The bioplastic films used for the degradation tests in liquid culture were prepared using the solvent-cast method [13]. First, 0.4 g of each plastic pellet was dissolved in 200 ml of chloroform and heated in a water bath at 60°C. After the pellets were completely dissolved, 50 ml of the solution was poured into a Petri dish, and the solvent was completely evaporated in a fume hood. The films were cut into 20 mg pieces, which were sterilized with 70%ethanol and UV irradiation on a clean bench. The prepared films were used for liquid culture, and the PBAT film was used for scanning electron microscopy (SEM) and gel permeation chromatography (GPC).

### Preparing Plates Containing Bioplastic

To prepare plates containing bioplastics, 1 g of bioplastic pellets were dissolved in 40 ml of DCM in a water bath at 60°C for 1 h. Once the pellets were completely dissolved, 100 ml of water and 2 ml of 2% Sarkosyl NL were added to the interface between the water and DCM. The mixture was sonicated using a Vibra-Cell VCX500 (Sonics & Materials, Inc., USA) with 15 s of pulsing at 30% amplitude for 10 min. Subsequently, a 1 g/l plastic emulsion uniformly dissolved in the aqueous phase of the solvent was added to Luria–Bertani broth (LB; Difco, USA) containing 10 g/l tryptone, 5 g/l yeast extract, and 5 g/l sodium chloride. All mixtures were sterilized by autoclaving for 15 min at 121°C. After sterilization, the autoclaved solution was poured into a Petri dish at an appropriate volume and cooled on a clean bench until solidification.

### Isolation and 16S rRNA Sequencing of PBAT-Degrading Microorganisms

Soil samples used in this study were collected from various environments. The compost was obtained from ABNEXO (Republic of Korea, 37.400563, 126.990730). Other samples were collected from the Soyang River (38.031293, 127.807195) on the shore of Jebudo, wastewater sludge, and a salt farm in Sinan (35.030707, 126.153760). Wastewater sludge was obtained from the Korea Institute of Industrial Technology. Approximately 0.5 g of soil sample was diluted with 1 ml of autoclaved distilled water, and the sample was multistage diluted to 10^-3^. The samples were respectively spread onto LB agar plates containing 0.1% PBAT emulsion for screening. After incubation for 3–5 days in a 42°C incubator, colonies forming clear zones were isolated from the LB-PBAT plates. The colonies were cultured in LB liquid media for 1 day, and stocks of each isolated microorganism were prepared containing 50% (w/v) glycerol and stored at −80°C for further use [13]. Nine different PBAT-degrading micro-organisms were used in this study.

Genomic DNA was extracted using the boiling method with Chelex resin, and PCR amplification was performed using the 27F primer, producing a 1.5 kb PCR product, which was subsequently purified and sequenced. Polymerase chain reaction (PCR) consisted of 1 μ of template DNA, 10 μ of hot-start green mix (consisting of Taq polymerase, dNTP, MgCl2, and buffer), universal primer 27F (AGA GTT TGA TCM TG CTC AG) and 1492 R (CGG TTA CCT TGT TAC GAC TT) 1 μ and 7 μ of water. PCR amplification was performed using a LifeTouch Thermal Cycler (Bioer Technology Co., Ltd., China). An initial preheating at 95°C for 3 min was followed by 35 cycles of 95°C for 30 s, 48°C for 39 s, and 72°C for 72s [14].

The obtained sequences were identified using the NCBI BLASTn database (https://blast.ncbi.nlm.nih.gov/Blast.cgi), and a phylogenetic tree was constructed using MEGA X software (https://www.megasoftware.net/). The phylogenetic tree in this study was constructed using the neighbor-joining (NJ) algorithm in MEGA X software. The evolutionary model was set to maximum composite likelihood, and 2,000 bootstrap replications were conducted to assess the reliability of the branches. Additionally, uniform rates among sites were assumed, and pairwise deletion was applied to handle gaps and missing data.

### Evaluation of Degradation Yield through Liquid Culture

The PBAT films used for the degradation tests in liquid culture were prepared. Before the primary culturing process, strains JO01, JO02, JO03, JO04, JO05, JO06, JY35, JY36, and JY49 were pre-cultured in 5 ml of approximate media with 1% inoculum of stock solution. Cultivation was performed at 30°C and 200 rpm for 16 h. In the main culture, pre-cultured cells were inoculated into 5 ml of approximate liquid medium in sterile flasks in a 14-ml round-bottom tube at 200 rpm for 7 days. To inoculate strains screened from marine samples, MB media was used, while LB media was used for the other strains. For comparison degradability with various strains, cultivation was carried out at various temperatures (20, 30, 37, and 42°C). All subsequent experiments using selected strains were performed at 30°C. The time-dependent degradation rate was measured using the same procedure over time (3, 5, 7, 10, 14, 21, and 28 days). Additionally, to find the optimal conditions, NaCl (1, 2, 3, 5, and 10%), various carbon sources (1%), and various nitrogen sources (1%) were added and cultivated for 1 week. After cultivation, the films in culture media were washed several times to remove the cells, media components, and water-soluble monomers. The collected residual PBAT films were lyophilized overnight to remove water from their surfaces. All experiments were performed in duplicate.

### Monitoring of Clear Zones by Solid Culture

To analyze and optimize the characteristics of the screened strain, clear-zone tests were conducted. The microorganism was pre-cultured in 5 ml of MB liquid media at 30°C and 200 rpm overnight. Paper discs (Toyo Roshi Kaisha, Japan) were then placed on the plates [15, 16], and 10 μl of the pre-cultured cells were inoculated onto the disc and incubated at 30°C for 14 days. The radii of the clear zones were measured by determining the distance between the paper disc and the edge of the clear zone.

### Esterase Activity Assay with *p*-Nitrophenyl Esters

To evaluate the esterase activity of the degrading strains, six *p*-nitrophenyl substrates (*p*-nitrophenyl acetate, *p*-nitrophenyl butyrate, *p*-nitrophenyl hexanoate, *p*-nitrophenyl octanoate, *p*-nitrophenyl decanoate, and *p*-nitrophenyl dodecanoate) were used. The enzyme reaction complex included 180 μl of 50 mM phosphate buffer (pH 7.4), 10 μl of supernatant (collected from the liquid culture centrifuged at 4°C and 13,000 ×*g* for 10 min), 5 μl of substrates dissolved in acetonitrile, and 5 μl of ethanol. The mixtures were reacted in an incubator at 37°C for 30 min. Absorbance was measured at 405 nm in a 96-well plate to confirm esterase activity [17].

### HPLC Analysis to Confirm PBAT Monomer Utilization Ability

*Bacillus* sp. JO01 was cultured in LB medium containing AA, TPA, and 1,4-butanediol to confirm its ability to utilize bioplastic monomers. To confirm the effects of the monomers on the degradation of the PBAT film, the monomers and films were incubated with *Bacillus* sp. JO01. The films and different concentrations of monomers were cultured in LB with 1% of *Bacillus* sp. JO01 at 30°C and 200 rpm. After 24, 48, and 72 h, 1 ml of the culture medium was transferred to an e-tube (1.5 ml) to measure the optical density and monomer utilization ability via HPLC. The culture medium was centrifuged at 4°C and 13,000 ×*g* for 15 min to obtain the supernatant containing the monomer of PBAT except cells. The supernatant was diluted 10-fold with HPLC water. The diluted solution was analyzed to confirm the residual monomer concentration using HPLC (Perkin Elmer, USA) equipped with a refractive index detector and a UV–Vis detector. To quantify acrylic acid (AA), terephthalic acid (TPA), and 1,4-butanediol, separation was performed using an Aminex HPX-87H column (300 × 7.8 mm internal diameter, Bio-Rad, USA). The flow rate of the mobile phase was maintained at 0.6 ml/min using 0.004 mol/l sulfuric acid (H_2_SO_4_), with analysis performed over 40 min in an isocratic elution method. Detection was carried out at a wavelength of 210 nm, and the oven temperature was set to 60°C during the operation [18,19].

### GC-FID Analysis

The residual amount of PBAT and the degradation yield were determined through GC–FID analysis, prior to which, fatty acid methyl ester derivatization was conducted to prepare the samples [20, 21]. For methanolysis of the samples, a mixture of 1 ml methanol/sulfuric acid (85:15 v/v) and 1 ml chloroform was added, and the vials were heated for 2 h at 100°C. The samples were subsequently cooled to room temperature, and 1 ml of HPLC-grade water was added to the vials, followed by vortexing for 1 min [22]. The organic phase layer at the bottom of the vials was then transferred to a 1.5-ml e-tube containing anhydrous sodium sulfate to eliminate residual water. Samples were filtered (0.2 μm pore size; Chromdisc, Republic of Korea) before injection into the GC–FID equipment. Filtered sample aliquots of 1 μl were then injected into a gas chromatograph (Young-lin 6500, Republic of Korea) operating in split mode (1/10). The chromatograph was equipped with a fused silica capillary column (Agilent HP-FFAP, 30 m × 0.32 mm, i.d. 0.25 μm film) and a flame ionization detector (FID). The inlet temperature was set at 210°C, and helium served as the carrier gas at a flow rate of 3 ml/min. The oven temperature followed a gradient program, starting from 80°C for 0–5 min, and reaching 220°C for 12–17 min. Throughout the experiments, the FID temperature remained constant at 230°C.

### Physical Analysis of PBAT films after Degradation

**SEM.** Surface changes in the PBAT films were analyzed using SEM. The films were then degraded by *Bacillus* sp. JO01 for 0, 3, 5, 7, and 10 days, and the residual films were collected. They were washed with distilled water to remove the medium components and cells and lyophilized. Subsequently, the PBAT films were coated with gold dust at 5 mA for 300 s, and backscattered electron images were obtained using a TM4000Plus SEM instrument (Hitachi, Japan) at a voltage of 5 kV [23].

**GPC.** The molecular weight changes in the degraded PBAT films were analyzed using gel permeation chromatography (YoungIn Chromass, Republic of Korea). To prepare the samples, the residual PBAT films were dissolved in 2 ml of chloroform and heated at 60°C for 1 h. The resulting solution was then filtered using a syringe filter (0.2 μm pore size; Chromdisc, Republic of Korea). The analysis was conducted using an HPLC apparatus, including a loop injector (Rheodyne 7725i), an isocratic pump with dual heads (YL9112), a column oven (YL9131), columns (Shodex, K-805, 8.0 mm I.D. × 300 mm; Shodex, K-804, 8.0 mm I.D. × 300 mm), and a refractive index detector (YL9170). A 60-μl sample was injected for analysis. Chloroform served as the mobile phase, with a flow rate of 1.0 ml/min and a temperature of 35°C. The data were processed using YL-Clarity software for a single YL HPLC instrument (YoungIn Chromass).

## Results

### Comparison of Degradability for Bioplastics and Selection of Superb Degrading Bacteria

**Fig. 1:**
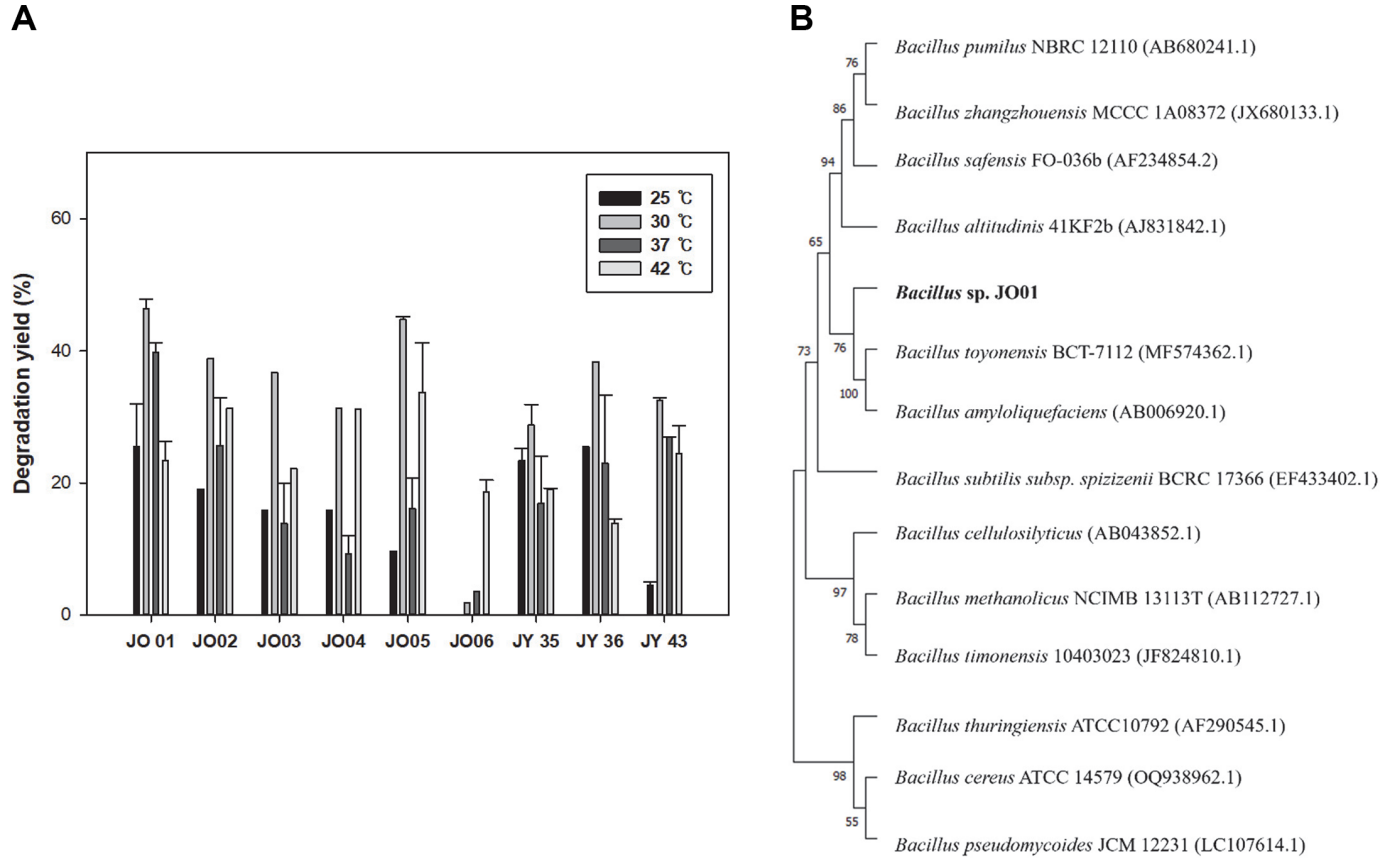
Comparison of degradation yield according to various strains. (**A**) Among the 9 strains, JO01 was shown the highest degradation yield at 30°C. The highest degradation yield was about 46% for 7 days. (**B**) Phylogenic tree of JO 01. The JO01 strain exhibiting high similarity (99%) with the *Bacillus toyonensis* BCT-7112.

To identify PBAT-degrading strains, a PBAT plate was prepared as described in the Materials and Methods section. Then, soil samples from five sites in South Korea were analyzed. Among the various strains that formed transparent hollows, those that formed significantly large transparent hollows were selected. To confirm and compare the degradability of PBAT by the strains using different methods, PBAT films were incubated with LB or MB at various temperatures. After 7 days of incubation, the amount of PBAT film in all samples decreased, indicating that the nine strains showed PBAT degradability (Fig. 1A). The bacterial strain showing the highest degradability and robustness to various temperatures was *Bacillus* sp. JO01. According to the 16S rRNA results, *Bacillus* sp. JO01 showed the highest similarity to *Bacillus toyonensis* BCT-7112 with 99% of similarity (Fig. 1B). *Bacillus toyonensis* is a gram-positive, spore-forming bacterium [24, 25] that produces PHA. Initially, *Bacillus* sp. JO01 was isolated at 42°C; however, based on the temperature optimization for degradation, it showed optimal growth and activity with 46% PBAT degradation at 30°C (Fig. 1A). All the following experiments were performed at 30°C.

### Evaluation of *Bacillus* sp. JO01 for PBAT Degradation

**Fig. 2:**
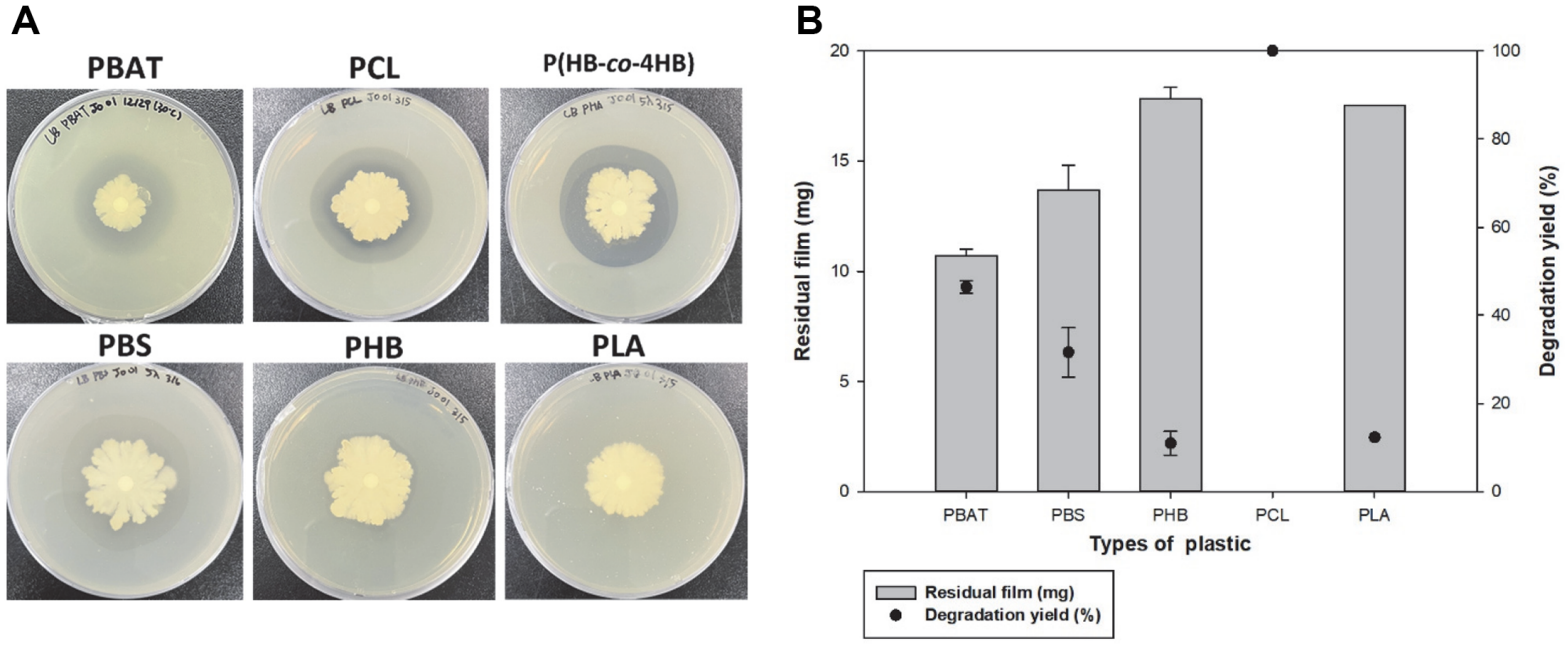
Comparision of the degradability for various bioplastics (**A**) Clear zone test of *Bacillus* sp. JO01, the clear zone was formed on PBAT, PCL, P(HB-co-4HB), PBS and PHB plate, (**B**) Degradation of plastic films by *Bacillus* sp. JO01. PCL and PBAT showed high degradation rateby *Bacillus* sp. JO01.

To compare the degradability of *Bacillus* sp. JO01 for the various types of bioplastics, a clear-zone test was conducted using LB agar plates containing PBAT, PCL, P(HB-*co*-4HB), PBS, PHB, and PLA. After 14 days of incubation, a clear zone was formed on all plastic plates, except for the PLA plate (Fig. 2A).

Liquid tests were performed to confirm the degradation activity of the five types of bioplastic films. *Bacillus* sp. JO01 had degradation activity not only for PBAT but for various other plastics, including PCL, PBS, and PHB (Fig. 2B). Liquid tests using PCL and PBAT showed high degradation yields after 7 days. In particular, for PCL, the degradation rate was almost 100% after 7 days. This indicates that *Bacillus* sp. JO01 can degrade homopolymers and copolymers, and moreover, that a single strain can be used to degrade various types of plastic.

**Fig. 3:**
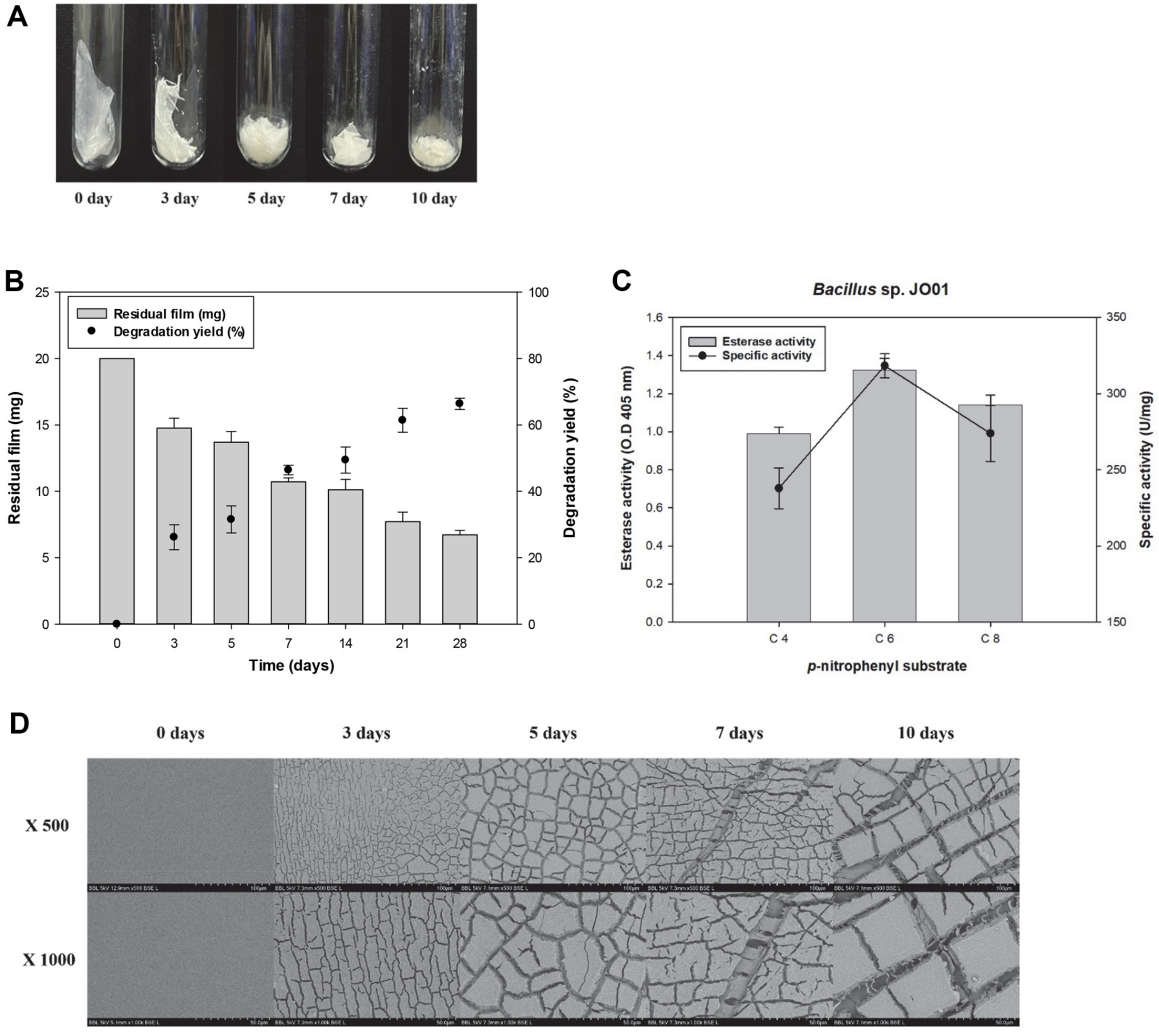
Evaluation of *Bacillus* sp. JO01 for PBAT degradation. (**A**) Image of degraded PBAT film by *Bacillus* sp. JO01, (**B**) PBAT degradation according to cultivation time. PBAT and the film was degraded by about 66% after 28 d. (**C**) Enzyme activity of *Bacillus* sp. JO01 (**D**) Changes in the surface of PBAT films after degradation.

Additionally, a liquid experiment was conducted for 28 days to monitor the time-dependent degradation of PBAT. Over time, the decrease in the film thickness was visually monitored (Fig. 3A). The PBAT film was reduced to approximately 14.5 mg after 3 days of incubation, with a degradation yield of around 25%. After 7 days, the degradation yield increased to about 46%. After 28 days, the PBAT film had degraded into visibly small fragments, with a degradation rate of approximately 66% (Fig. 3B). While many studies have investigated the degradation of PBAT in blends with PLA [26-28], relatively few have explored PBAT degradation alone. Previous studies that tested PBAT alone reported average degradation rates of only 4–10%, whereas *Bacillus* sp. JO01 demonstrated a relatively high degradation rate for PBAT [9-11]. This indicates that microbial species with efficient enzymatic systems may play a key role in accelerating the degradation process. Microorganisms capable of degrading organic materials often rely on hydrolytic enzymes, such as esterases and lipases [29]. Among them, Bacillus strains stand out for their ability to thrive across a wide range of pH and temperature conditions, maintaining enzymatic activity over extended periods [30]. This environmental adaptability, along with their secretion of hydrolytic enzymes, makes Bacillus strains particularly effective in degrading various substrates, including PBAT [31]. To assess the enzymatic activity of *Bacillus* sp. JO01, *p*-nitrophenyl esters were used. The specific activities for C4, C6, and C8 substrates were measured at 237.82, 318.35, and 273.92 U/mg, respectively (Fig. 3C). One unit (U) is defined as the amount of enzyme that catalyzes the reaction of 1 μmol of substrate per minute [32].

### Physical Properties of PBAT Films after Degradation

After confirming the degradability of *Bacillus* sp. JO01, changes in the surface of the PBAT film were monitored using SEM. The degraded samples were recovered on days 3, 5, 7, and 10. Compared to the film before degradation, cracks were observed after 3 days of degradation. The cracks on the degraded film became larger as the incubation time increased (Fig. 3D).

In addition, changes in the molecular weight of the degraded PBAT films were measured by GPC. The PBAT films were degraded for 3, 5, 7, and 10 days under liquid conditions and recovered to determine the number-average molecular weight (Mn), weight-average molecular weight (Mw), and polydispersity index (PDI) using GPC (Table 2). The molecular weight of a polymer changes because of molecular chain destruction or rearrangement, which is another significant change in the chemical structure of the material during polymer degradation [33]. Compared to the film on day 3, the film after 14 days showed decreases in Mw and Mn from 14.31 × 10^4^ to 5.56 × 10^4^, and from 8.08 × 10^4^ to 3.95 × 10^4^. Over time, all the values tended to decrease, suggesting that the degree of degradation of the PBAT film by *Bacillus* sp. JO01 gradually increased. Furthermore, the PDI initially increased and then gradually decreased (Table 2). Normally, during the degradation process, polymer chains break down into smaller fragments that can occur in different ways, thereby influencing the PDI value. If the degradation process causes random chain scission, the molecular weight distribution broadens because both high-molecular-weight polymers break into a wide range of smaller fragments. This could lead to an increase in the PDI [34]. In addition, if the fragments become more uniform in size, the PDI may stabilize or decrease slightly [35]. Considering that other reports from our group showed that cutting the ends of plastic polymers, rather than the inside, did not significantly affect the molecular weight distribution [36], the breakage of PBAT by *Bacillus* sp. JO01 seemed to be an exo-type, and more enzymatic studies will be needed to determine the function of the enzymes.

### Evaluation of Nutrient Condition for PBAT Degradation by *Bacillus* sp. JO01

Previous studies have shown that the optimum temperature for PBAT degradation by *Bacillus* sp. JO01 is 30°C. Tests were conducted in various cultivation environments to determine the optimal conditions for PBAT degradation. Microorganisms generally use carbon sources, nitrogen sources, and salts as nutrients, and sometimes their addition causes more cells to grow [37]. Seven carbon sources were added for film degradation (Fig. S1). Compared with the sample without a carbon source, the degradation yield of the samples with carbon sources decreased. It is not beneficial to produce esterases that are rich in carbon sources.

Four nitrogen sources were tested to confirm their effects on PBAT degradation (Fig. S1). The addition of nitrogen sources positively affected the PBAT degradation. In particular, when (NH_4_)_2_SO_4_ was added, the decomposition rate increased to around 62%. Ammonium sulfate ((NH_4_)_2_SO_4_) provided a readily available source of nitrogen, which was a crucial nutrient for bacterial growth. Nitrogen is a fundamental component of amino acids, proteins, nucleic acids, and other cellular components [38]. *Bacillus* species, like many other bacteria, require nitrogen to synthesize these essential molecules [39]. In addition, ammonium sulfate enhances the activity of certain enzymes. For instance, it can stimulate the activity of enzymes involved in nitrogen metabolism, thereby promoting efficient nutrient utilization and growth.

Finally, the effect of salt on the degradation was tested. By adding different concentrations of NaCl, the decomposition rate decreased as the concentration increased (Fig. S1). This result indicates that *Bacillus* sp. JO01 had little or no resistance to salt, which we expected due to the fact that *Bacillus* sp. JO01 was found in soil and compost, but not in the marine sample. These results confirm that PBAT degradation by *Bacillus* sp. JO01 showed the highest efficiency when there was an additional nitrogen source.

### Effect of Monomer for Degradation PBAT by *Bacillus* sp. JO01

Unlike biomass-based plastics, which use biological components, PBAT is a petroleum-based, chemically synthesized bioplastic. Thus, the mechanism of PBAT degradation by microorganisms may differ from that of some bioplastics used as carbon sources in microbial metabolism [9]. PBAT is composed of adipic acid (AA), terephthalic acid (TPA), and 1,4-butanediol (BDO). TPA monomers are responsible for the rigid domain, whereas BDO, together with the AA monomer, controls the polymer flexibility. Among these compounds, TPA, an aromatic monomer, is difficult for microorganisms to degrade [8]. Therefore, we conducted consumption tests for AA, TPA, and BDO, which are the monomers of PBAT, and their effects on plastic film degradation.

**Fig. 4:**
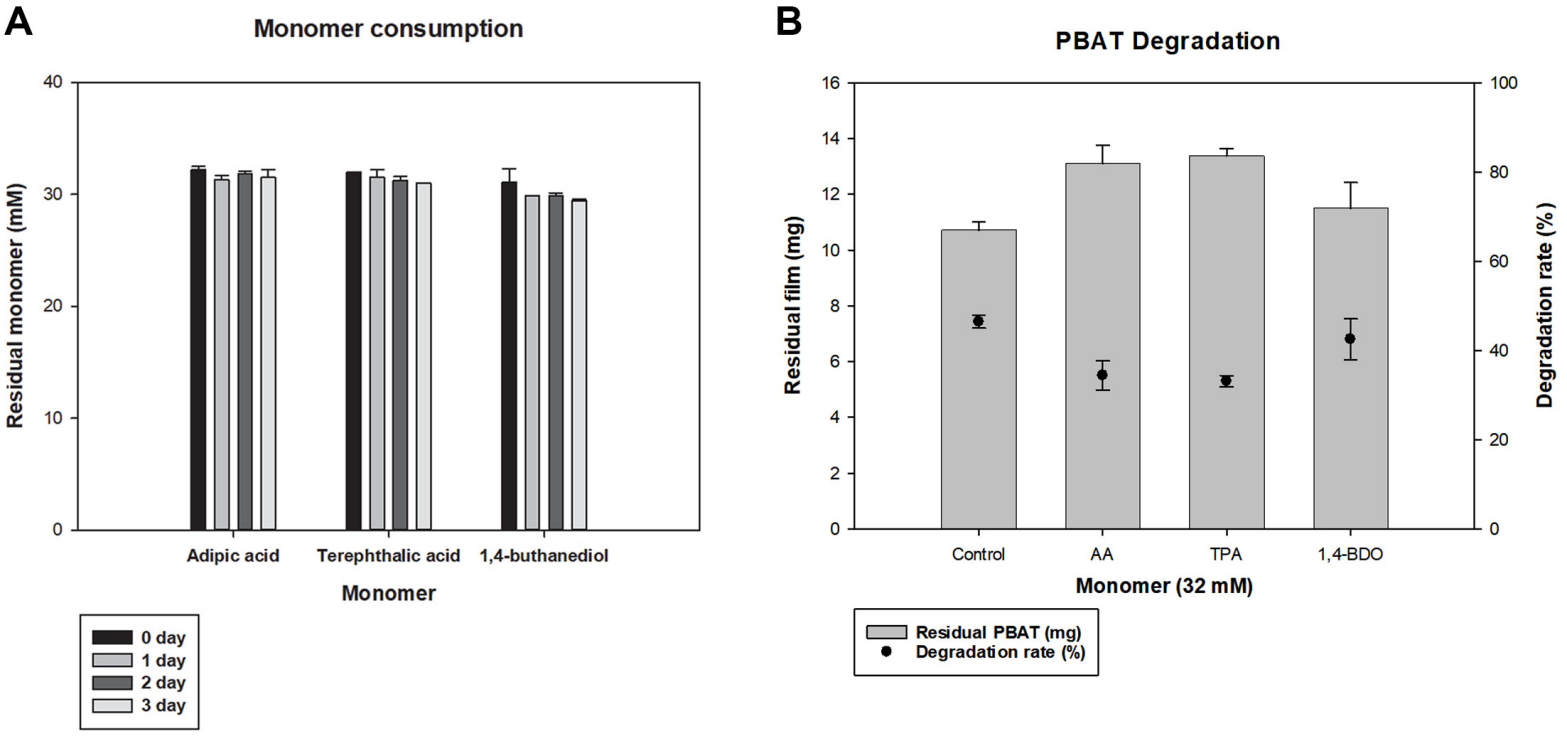
Monomer consumption (**A**) Consumption by *Bacillus* sp. JO01 for each of the 32 mM monomers, monomers were not decreased after cultivation. (**B**) PBAT degradation according to addition of monomer (32 mM), when each monomer was added, the degradation rate was decreased.

To confirm the monomer consumption by *Bacillus* sp. JO01, HPLC analysis was performed (Fig. 4A). Observation of each monomer and strain up to day 3 of incubation showed that the monomer did not decrease, which indicated that *Bacillus* sp. JO01 did not consume monomers.

**Fig. 5:**
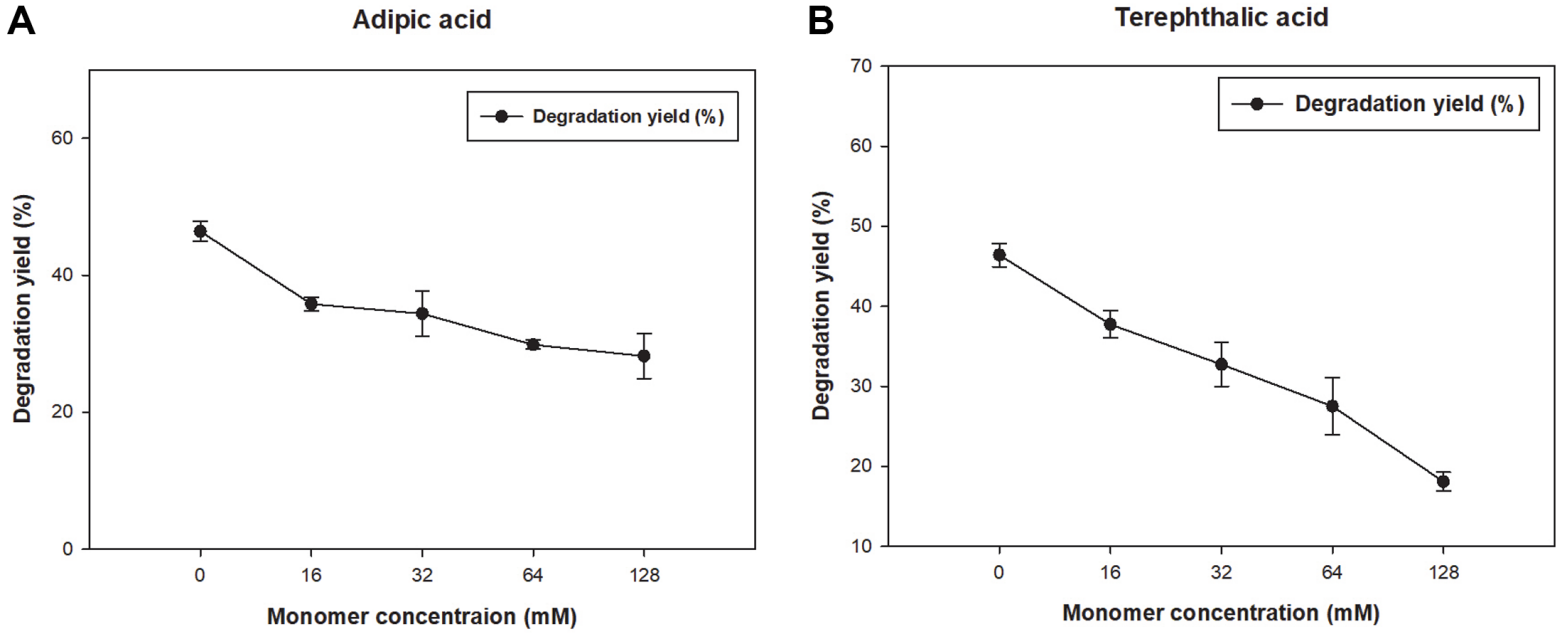
Effect on degradation of monomers PBAT degradation according to addition of monomer (**A**) Comparison of PBAT film degradation by concentration of (**A** and **B**) comparison of PBAT film degradation by concentration of TPA.

When the inhibitory effect was compared with that of control, which had PBAT film and *Bacillus* sp. JO01, and other samples with PBAT film and *Bacillus* sp. JO01 and 32 mM of monomers (Fig. 4B), we found that the addition of AA, TPA, and BDO showed 74.12%, 67.87%, and 98.55% relative degradation to control respectively. This suggested that AA and TPA inhibited PBAT degradation significantly. Subsequently, the effect of the monomers on film degradation was monitored, and each monomer was added at various concentrations (16–128 mM). Therefore, various concentrations of AA, TPA, and PBAT films were incubated together, and as the concentration increased, the degradation rate decreased (Fig. 5A and 5B). Unlike TPA, which is difficult for microorganisms to approach and degrade because of its high crystallinity and hydrophobicity, AA acts as a PBAT film inhibitor [40]. The 30% inhibition of PBAT degradation was 16 mM for AA and 32 mM for TPA. Considering that 16 mM AA was equivalent to the monomer from 5.8 mg of PBAT film, we expected that even if a small amount was added, degraded monomers, such as AA and TPA, could inhibit the degradation of PBAT once they were not consumed by microbes.

**Fig. 6:**
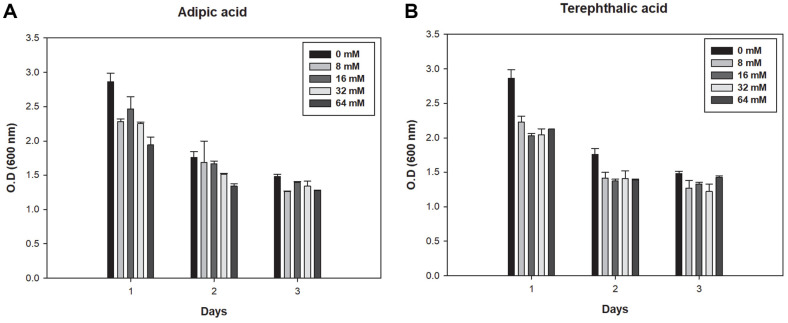
Effects of monomers on cell growth (**A**) Cell growth according to concentration of adipic acid, (**B**) Cell growth according to concentration of terephthalic acid. Cell growth decreased when each monomer was added.

Enzymatic activity and the number of cells could affect plastic degradation by microorganisms [41]. As shown in Fig. 6, when each monomer was added, the cell growth rate decreased compared with that of the control (0 mM). In particular, the cell growth rate was significantly reduced when the monomer compared to the control was added at the beginning of the culture. When AA and TPA were added at 64 mM, it was confirmed that cell growth was reduced by 32.1% and 25.6% compared to the control, respectively. Therefore, the monomers acted as inhibitors because film degradation was suppressed as the number of cells acting on the plastic decreased with the addition of monomers. Interestingly, the IC_30_ of AA and TPA was 57.8 mM and 17.24 mM, suggesting that AA showed higher inhibitory effect on the activity of degrading enzyme and TPA showed higher inhibitory effect on the growth of *Bacillus* sp. JO01. Overall, biodegradation and consumption should be considered in microbial degradation during bulk degradation processes.

## Discussion

Considering the increased use of bioplastics, the discovery of novel, plastic-degrading microbes is crucial. In particular, PBAT, known for its flexibility and processability, offers an interesting target for plastic-degrading microbes. This study demonstrated that a novel strain, *Bacillus* sp. JO01, has excellent ability to degrade PBAT films at 30°C, resulting in about 66% degradation after 28 days.

Although *Bacillus* sp. JO01 exhibits strong enzyme production capabilities for PBAT degradation, the presence of monomers, such as AA, 1,4-butanediol, and TPA, was found to inhibit the cell growth of the bacteria, thereby reducing degradation efficiency. Specifically, 16 mM of AA significantly suppressed bacterial growth, inhibiting the hydrolysis of PBAT. This finding highlights the challenge of maintaining optimal microbial activity in the presence of inhibitory by-products, which must be addressed to enhance the overall degradation process.

One potential solution to these challenges involves a two-stage degradation strategy. In the first stage, microbial growth is promoted to generate sufficient biomass, ensuring robust microbial activity. In the second stage, PBAT is introduced into the culture medium, allowing the microorganisms to focus on degradation. This sequential approach could mitigate the inhibitory effects of toxic monomers during the initial growth phase, ultimately improving overall degradation efficiency.

Additionally, UV or plasma pretreatments could further improve efficiency by inducing oxidation and breaking polymer chains, making PBAT more accessible to microbes [42, 43]. These promising approaches could enable future research to overcome the challenges associated with PBAT degradation and optimize bioplastic degradation systems.

This study highlighted the potential of *Bacillus* sp. JO01 and provides key insights for optimizing microbial degradation systems. Considering that many bacteria only produce hydrolyzing enzymes and do not utilize monomers [6], these results clearly show the need for both the production of hydrolases and the utilization of monomers. Although this can be solved with a consortium of bacteria in nature [44], a consolidated system for the fast degradation of bioplastics should be considered.

## Supplemental Materials

Supplementary data for this paper are available on-line only at http://jmb.or.kr.



## Figures and Tables

**Table 1:** Previous reports of PBAT degradation by a single strain of bacteria.

Bacteria	Temperature	Days of degradation	Degradation yield (%)	Reference
*Stenotrophomonas* sp. YCJ1	37°C	5	10.14	[9]
*Peribacillus frigoritolerans* S2313	28°C	7	3.98	[10]
*Bacillus* sp. SUST B_2_	37°C	12	10.5	[12]
*Bacillus pumilus*	30°C	10	6	[11]
*Bacillus* sp. JY35	30°C	21	50	[13]
*Bacillus* sp. JO01	30°C	21	61	In this study

**Table 2:** Molecular weight of degraded PBAT in liquid culture analyzed via gel permeation.

Day	Mn × 10^4^	Mw × 10^4^	PDI (Mw/Mn)
3	8.08	14.31	1.53
5	6.81	13.54	1.61
7	6.57	12.74	1.60
10	5.10	8.39	1.44
14	3.95	5.56	1.33

## References

[1] Geyer R, Jambeck JR, Law KL (2017). Production, use, and fate of all plastics ever made. Sci. Adv..

[2] Bhuwal AK, Singh G, Aggarwal NK, Goyal V, Yadav A (2013). Isolation and screening of polyhydroxyalkanoates producing bacteria from pulp, paper, and cardboard industry wastes. Int. J. Biomater..

[3] Lucas N, Bienaime C, Belloy C, Queneudec M, Silvestre F, Nava-Saucedo JE (2008). Polymer biodegradation: mechanisms and estimation techniques - A review. Chemosphere.

[4] Alshehrei F (2017). Biodegradation of synthetic and natural plastic by microorganisms. J. Appl. Environ. Microbiol..

[5] Accinelli C, Saccà ML, Mencarelli M, Vicari A (2012). Deterioration of bioplastic carrier bags in the environment and assessment of a new recycling alternative. Chemosphere.

[6] Danso D, Chow J, Streita WR (2019). Plastics: environmental and biotechnological perspectives on microbial degradation. Appl. Environ. Microbiol..

[7] Ratshoshi BK, Farzad S, Görgens JF (2024). A techno-economic study of Polybutylene adipate terephthalate (PBAT) production from molasses in an integrated sugarcane biorefinery. Food Bioprod. Processing.

[8] Boll M, Geiger R, Junghare M, Schink B (2020). Microbial degradation of phthalates: biochemistry and environmental implications. Environ. Microbiol. Rep..

[9] Jia H, Zhang M, Weng Y, Zhao Y, Li C, Kanwal A (2021). Degradation of poly(butylene adipate-*co*-terephthalate) by *Stenotrophomonas* sp. YCJ1 isolated from farmland soil. J. Environ. Sci. (China).

[10] Wufuer R, Li W, Wang S, Duo J (2022). Isolation and degradation characteristics of PBAT film degrading bacteria. Int. J. Environ. Res. Public Health.

[11] Muroi F, Tachibana Y, Soulenthone P, Yamamoto K, Mizuno T, Sakurai T (2017). Characterization of a poly(butylene adipate-coterephthalate) hydrolase from the aerobic mesophilic bacterium *Bacillus pumilus*. Polym. Degrad. Stab..

[12] kanwal A, Zhang M, Sharaf F, Chengtao L (2022). Screening and characterization of novel lipase producing *Bacillus* species from agricultural soil with high hydrolytic activity against PBAT poly (butylene adipate co terephthalate) co-polyesters. Polym. Bull..

[13] Cho JY, Park SL, Kim SH, Jung HJ, Cho DH, Kim BC (2022). Novel Poly(butylene adipate-co-terephthalate)-degrading *Bacillus* sp. JY35 from wastewater sludge and its broad degradation of various bioplastics. Waste Manag..

[14] Shin N, Oh J, Kim S, Lee Y, Shin Y, Choi S (2024). Dual application of p-nitrophenol alkanoate-based assay for soil selection and screening of microbial strains for bioplastic degradation. J. Microbiol. Biotechnol.

[15] Bhatia SK, Yoon JJ, Kim HJ, Hong JW, Gi Hong Y, Song HS (2018). Engineering of artificial microbial consortia of *Ralstonia eutropha* and *Bacillus subtilis* for poly(3-hydroxybutyrate-co-3-hydroxyvalerate) copolymer production from sugarcane sugar without precursor feeding. Bioresour. Technol..

[16] Shin N, Kim SH, Cho JY, Hwang JH, Kim HJ, Oh SJ (2023). Fast degradation of polycaprolactone/poly(butylene adipate-coterephthalate) blends by novel Bacillus strain NR4 with broad degrading activity. J. Polym. Environ..

[17] Kim SH, Cho JY, Nara-Shin, Hwang JH, Kim HJ, Oh SJ (2023). Revealing the key gene involved in bioplastic degradation from superior bioplastic degrader *Bacillus* sp. JY35. Int. J. Biol. Macromol..

[18] Yang SY, Choi TR, Jung HR, Park YL, Han YH, Song HS (2020). Development of glutaric acid production consortium system with α-ketoglutaric acid regeneration by glutamate oxidase in *Escherichia coli*. Enzyme Microb. Technol..

[19] Lee SM, Lee HJ, Kim SH, Suh MJ, Cho JY, Ham S (2021). Screening of the strictly xylose-utilizing *Bacillus* sp. SM01 for polyhydroxybutyrate and its co-culture with *Cupriavidus necator* NCIMB 11599 for enhanced production of PHB. Int. J. Biol. Macromol..

[20] Sathiyanarayanan G, Bhatia SK, Song HS, Jeon JM, Kim J, Lee YK (2017). Production and characterization of medium-chainlength polyhydroxyalkanoate copolymer from Arctic psychrotrophic bacterium *Pseudomonas* sp. PAMC 28620. Int. J. Biol. Macromol..

[21] Bhatia SK, Gurav R, Choi TR, Han YH, Park YL, Park JY (2019). Bioconversion of barley straw lignin into biodiesel using *Rhodococcus* sp. YHY01. Bioresour. Technol..

[22] Park YL, Bhatia SK, Gurav R, Choi TR, Kim HJ, Song HS (2020). Fructose based hyper production of poly-3-hydroxybutyrate from *Halomonas* sp. YLGW01 and impact of carbon sources on bacteria morphologies. Int. J. Biol. Macromol..

[23] Jung HR, Choi TR, Han YH, Park YL, Park JY, Song HS (2020). Production of blue-colored polyhydroxybutyrate (PHB) by onepot production and coextraction of indigo and PHB from recombinant *Escherichia coli*. Dyes Pigm..

[24] Ratnaningrum D, Saraswaty V, Priatni S, Lisdiyanti P, Purnomo A, Pudjiraharti S (2019). Screening of polyhydroxyalkanoates (PHA)-producing bacteria from soil bacteria strains, IOP Conference Series: Earth and Environmental Science.

[25] R Wan Abdul Razak W, J Mohamad Yusuf N, Abdul-Aziz A, K Navaratnam S, Zubir I, E Rizlan Ross E (2019). Screening and isolation of polyhydroxyalkanoates (PHA)-producing bacteria from landfill by using Cocoa Pod Husks as carbon source. Int. J. Eng. Technol..

[26] Xu Z, Zheng B, Yang Y, Yang Y, Jiang G, Tian Y (2024). Effects of biodegradable (PBAT/PLA) and conventional (LDPE) mulch film residues on bacterial communities and metabolic functions in different agricultural soils. J. Hazard. Mater..

[27] Munhoz DR, Meng K, Wang L, Lwanga EH, Geissen V, Harkes P (2024). Exploring the potential of earthworm gut bacteria for plastic degradation. Sci. Total Environ..

[28] Fu Y, Wu G, Bian X, Zeng J, Weng Y (2020). Biodegradation behavior of poly(Butylene Adipate-Co-Terephthalate) (PBAT), poly(Lactic Acid) (PLA), and their blend in freshwater with sediment. Molecules.

[29] Roohi, Bano K, Kuddus M, Zaheer MR, Zia Q, Khan MF (2017). Microbial enzymatic degradation of biodegradable plastics. Curr. Pharm. Biotechnol..

[30] Bernardeau M, Lehtinen MJ, Forssten SD, Nurminen P (2017). Importance of the gastrointestinal life cycle of Bacillus for probiotic functionality. J. Food Sci. Technol..

[31] Guncheva M, Zhiryakova D (2011). Catalytic properties and potential applications of Bacillus lipases. J. Mol. Catal. B Enzym..

[32] Zhu B, Ye Q, Seo Y, Wei N (2022). Enzymatic degradation of polyethylene terephthalate plastics by bacterial curli display PETase. Environ. Sci. Technol. Let.t.

[33] Tertyshnaya YV, Podzorova MV, Khramkova AV, Ovchinnikov VA, Krivandin AV (2023). Structural rearrangements of polylactide/natural rubber composites during hydro- and biotic degradation. Polymers (Basel).

[34] Bo L, Guan T, Wu G, Ye F, Weng Y (2022). Biodegradation behavior of degradable mulch with poly (Butylene Adipate-co-Terephthalate) (PBAT) and poly (Butylene Succinate) (PBS) in simulation marine environment. Polymers (Basel).

[35] Cho JY, Lee Park S, Lee HJ, Kim SH, Suh MJ, Ham S (2021). Polyhydroxyalkanoates (PHAs) degradation by the newly isolated marine *Bacillus* sp. JY14. Chemosphere.

[36] Kim SH, Cho JY, Cho DH, Jung HJ, Kim BC, Bhatia SK (2022). Acceleration of polybutylene succinate biodegradation by *Terribacillus* sp. JY49 isolated from a marine environment. Polymers (Basel).

[37] Ruiz B, Chávez A, Forero A, García-Huante Y, Romero A, Snchez M (2010). Production of microbial secondary metabolites: regulation by the carbon source. Crit. Rev. Microbiol..

[38] Dev S, Patra AK, Mukherjee A, Bhattacharya J (2015). Suitability of different growth substrates as source of nitrogen for sulfate reducing bacteria. Biodegradation.

[39] Abdel-Mawgoud AM, Aboulwafa MM, Hassouna NAH (2008). Optimization of surfactin production by *Bacillus subtilis* isolate BS5. Appl. Biochem. Biotechnol..

[40] Yoshida S, Hiraga K, Takehana T, Taniguchi I, Yamaji H, Maeda Y (2016). A bacterium that degrades and assimilates poly(ethylene terephthalate). Science.

[41] Rana KI (2019). Usage of Potential Micro-organisms for Degradation of Plastics. Open Journal of Environmental Biology.

[42] Falkenstein P, Gräsing D, Bielytskyi P, Zimmermann W, Matysik J, Wei R (2020). UV pretreatment impairs the enzymatic degradation of polyethylene terephthalate. Front. Microbiol..

[43] Ji SH, Seok DC, Yoo S (2023). Improved biodegradability of low-density polyethylene using plasma pretreatment and plasticdegrading bacteria. Environ. Technol. Innov..

[44] Bhatia SK, Bhatia RK, Choi YK, Kan E, Kim YG, Yang YH (2018). Biotechnological potential of microbial consortia and future perspectives. Crit. Rev. Biotechnol..

