# Supplementary material for: Discovery of a Novel Bacillus sp. JO01 for the Degradation of Poly(butylene adipate-co-terephthalate)(PBAT) and Its Inhibition by PBAT Monomers
Source: J Microbiol Biotechnol. 2024 Nov 25;35:e2408051. doi: 10.4014/jmb.2408.08051 (PMC11813363; doi:10.4014/jmb.2408.08051)
Supplement: Supplementary file 1 [file jmb-35-e2408051-supple.pdf]

J. Microbiol. Biotechnol. 2025. 35: e2408051

https://doi.org/10.4014/jmb.2408.08051

Supplementary Figure

Fig. S1. Optimization of PBAT degradation by JO 01. (A) 7 carbon sources were used with JO01 for PBAT degradation. **(B)** 4 nitrogen sources were used with JO01 for PBAT degradation. **(C)** Degradation yield according to salt concentration at 30℃

(A)

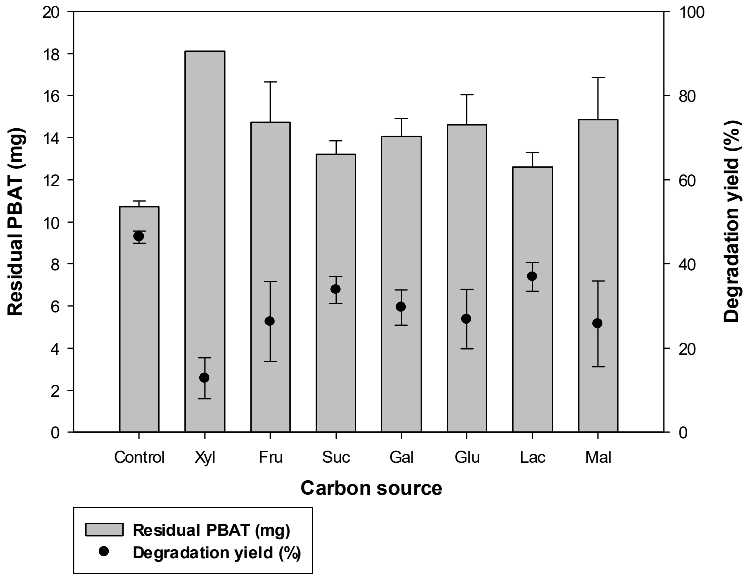

(B)

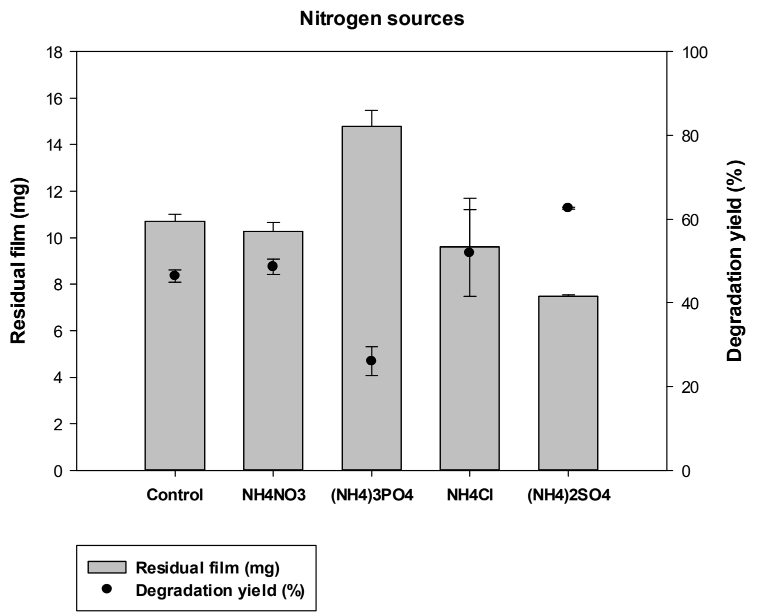
Nitrogen sources
Residual film (mg)
Degradation yield (%)
0
2
4
6
8
10
12
14
16
18
0
20
40
60
80
100
Control
NH4NO3
(NH4)3PO4
NH4Cl
(NH4)2SO4
Residual film (mg)
Degradation yield (%)

(C)

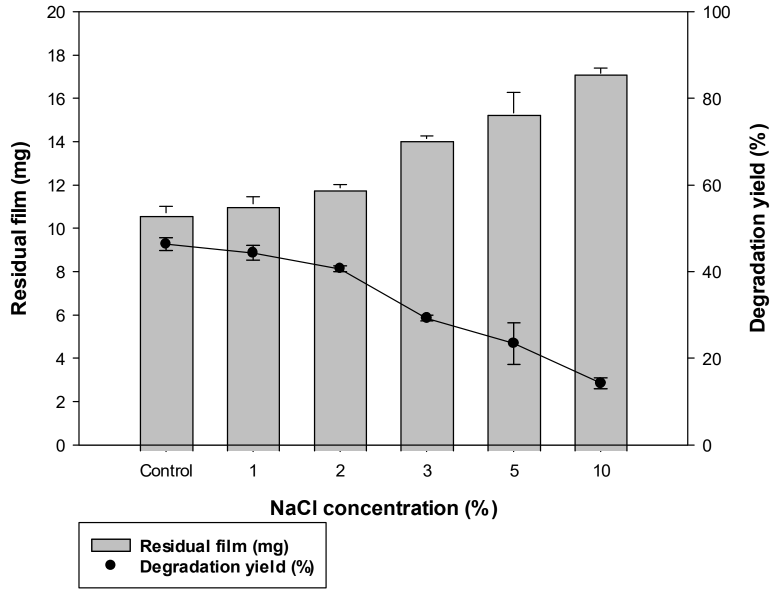
Residual film (mg)
Degradation yield (%)
0
2
4
6
8
10
12
14
16
18
20
0
20
40
60
80
100
Control
1
2
3
5
10
NaCl concentration (%)
Residual film (mg)
Degradation yield (%)
